# Factors affecting self-esteem and disease acceptance in patients from infertile couples

**DOI:** 10.3389/fpubh.2023.1177340

**Published:** 2023-07-14

**Authors:** Małgorzata Nagórska, Barbara Zych, Bogdan Obrzut, Dorota Darmochwał-Kolarz

**Affiliations:** ^1^Institute of Medical Sciences, Medical College of Rzeszow University, Rzeszow, Poland; ^2^Institute of Health Sciences, Medical College of Rzeszow University, Rzeszow, Poland

**Keywords:** infertility, self-esteem, disease acceptance, SES, AIS

## Abstract

**Introduction:**

Infertility has been diagnosed in millions of people around the world and is described as a complex medical, mental, and social problem that affects many aspects of life. The aim of the study was to extract the determining factors and the level of self-esteem and the degree of acceptance of the disease in infertile patients and to find differences between women and men in this aspect.

**Methods:**

A total 456 patients (235 women and 221 men) from infertile couples participated in a cross-sectional study. To collect data a Personal Information Form (PIF), Rosenberg Self-Esteem Scale (SES), and Acceptance of Illness Scale (AIS) were used.

**Results:**

The overall self-esteem score for the whole sample was 30.50 (15 ± 30) points and for acceptance of the disease 32.4 (8 ± 40) points. In the study group, men obtained a slightly higher level of self-esteem than women (31.00 vs. 30.04 points). Additionally, men had a higher level of acceptance of the disease (33.12 vs. 31.80) than women. Socio-demographic factors such as age and level of education had impact on scores SES and AIS. Clinical factors did not determine the results of SES and AIS, both in the overall sample and in the female and male groups.

**Conclusion:**

Self-esteem in patients from infertile couples increases with age and level of education. There are also significant differences between women and men, i.e., positive correlations between the level of education and self-esteem in men and the degree of acceptance of the disease in women.

## 1. Introduction

Infertility was described by World Health Organization (WHO) as a disease of male or female reproductive system defined by an inability to achieve a clinical pregnancy after 12 months of regular sexual intercourse without using contraception. A couple is considered infertile if they have been diagnosed with infertility as defined by the WHO. Infertility should not be misunderstood with sterility. Sterility is inability to naturally conceive and produce a live child by a couple (1). Estimated data shows that that this issue concerns 186 million individuals worldwide (2). Polish Society of Reproductive Medicine and Embryology (PSRME) and the Polish Society of Gynecologists and Obstetricians (PSGO) estimated data confirms that in Poland the problem with fertility affects about 1 million couples (3, 4).

The difficult situation faced by infertile couple results in the failure to meet one of the basic needs of most people, that of having children. Being a parent is an individual desire, but also a social expectation. Infertility from a social perspective is the inability to achieve the desired social role of parenthood, which is why it is often associated with psychological stress (5).

In modern societies, parenthood is postponed until later, and most people assume that they can become parents when they decide to do so (6, 7). Therefore, the problem of conceiving a child and the diagnosis of “infertility” is usually a big shock for the couple, this is associated with an uncertain prognosis and is a source of stress that can impact the wellbeing of people with fertility disorders (8–15).

Diagnosing and treating infertility is fraught and takes a long time with a relatively low success rate (about 20–40%) (16), which is also a source of stress and frustration (16–19). It is confirmed that there are gender differences between women and men in the approach to diagnosis together with the treatment of infertility. The women are more exposed during treatment and report stronger reactions both to infertility and treatment overall (20). For this reason, infertile women are more burdened by infertility stress and had stronger emotional reactions to infertility when compared to the men (21).

According to literature, infertility studies predominantly focus on women (22, 23). Many authors also confirm that there are differences between women and men and types of concerns in areas such as physical health, mental health, relationship satisfaction and satisfaction with sex life, social stigma or attitudes toward illness. Many studies have shown that women generally have less tolerance for accepting infertility in relationships. General health problems are more common in infertile women than men (24). With regards to mental health, women also had higher levels of stress (25, 26) and higher severity of depressive symptoms than men from infertile couples (27–29). Men in infertile couples had greater feeling of satisfaction from relationship and greater satisfaction with sexual life than woman (25, 30). There are also communication differences between men and women in infertile relationships. Women are more open to talking about infertility, and they more frequently address this topic with their partners, friends and family. Men, on the other hand, are reluctant to talk about infertility and tend to limit conversations to their partner or medical staff. Regardless of this, partners are the greatest support for each other in this situation (31, 32). It was also confirmed that infertile couples often experience social stigma and this issue is more likely to affect women (24, 33–35). In women, self-stigma also occurs, and this has bad impact on their psychosocial functioning and self-esteem (36). Other sources confirm that men are usually more optimistic than women in the situation of infertility (27, 32).

The experience of infertility affects many aspects of life and leads to a crisis for many couples (8, 37). Knowing that it is impossible to have a child initially means emotional shock, sadness and disappointment for the couple (32). This situation often leads to a sense of frustration, self-criticism, guilt, a decrease in self-esteem (38–42), which makes it difficult to accept the situation and come to terms with the diagnosis. In our work, we focus on determining the level of self-esteem and acceptance of the disease in infertile patients.

Self-esteem is a kind of general self-evaluation. According to Rosenberg, self-esteem is a positive or negative attitude toward oneself and a general evaluation of one’s own thoughts and feelings in relation to oneself (43). Self-esteem is an indicator of well-being because it has a positive relationship with mental health, social adjustment, and quality of life (44–46).

Self-esteem can change both in the short- and long-term perspective and depends on different events and situations in life (47), e.g., Cox et al. pointed out that self-esteem in women increases after successful infertility treatment (48). Long-term infertility is usually associated with the occurrence of negative emotions and psychological consequences, such as chronic stress and anxiety (49). Many authors confirm self-esteem is lower in infertile men and women, comparing to a healthy population (39–41).

In our study we tried to also identify acceptance of the disease, what should be understood as the absence of problems with adaptation to the limitations resulting from the disease, a sense of independence and self-sufficiency, and unreduced self-esteem (50, 51). Patients who accept their illness are more optimistic and hopeful, they have more trust in the proposed treatment, and they also more actively take part in the therapy (52). The level of acceptance of the disease depends on the nature of the disease and the discomfort it creates, as well as on socio-demographic conditions (50, 51). The acceptance of the disease in the case of infertility varies and depends on gender, prognosis (52) and the scale of social support and the economic situation of the population (49).

As Dembinska mentioned the acceptance of one’s own infertility is much less often described in the literature compared to other chronic diseases (50). To our knowledge in Poland there has only been one study on self-esteem and the level of acceptance of the disease and concerned only infertile women. Our study covers both sexes and was designed to establish the factors determining the level of self-esteem and the degree of acceptance of infertility in patients from infertile couples and to find differences between women and men in this aspect.

## 2. Materials and methods

### 2.1. Participants and course of the study

Quantitative, non-experimental method of empirical research was used. It was a cross sectional study. After obtaining permission from medical facilities and the ethical commission 500 patients were invited to take part who met the conditions for inclusion to the study. Detailed criteria inclusion/exclusion are presented in Table 1. The estimate data for Poland was considered to calculate the estimate data (approx. 1 milion couples). The sample size was calculated using the G*Power 3.1.9.2 program (Faul, F., Erdfelder, E., Lang, A.-G., Buchner, A., Düsseldorf, Germany). The minimum sample size was 383. Total 456 men and women from infertile couples took part in our study.

**Table 1:** The main eligibility criteria for study group.

Inclusion criteria	Exclusion criteria
Confirmed diagnosis infertility in couple	Couples without confirmed diagnosis of infertility
Age ≥ 18 years old	Age < 18 years old
No communication problems and the ability to understand and fill in the questionnaire	Communication problems and lack of ability to understand and fill in the questionnaire
Voluntary consent to participate in the study	Lack of consent to participate in the study

The research was conducted in three medical facilities offering infertility treatment in south-eastern Poland. Before conducting the project, we got a written permission from all medical facilities and from a relevant ethical committee. Patients who had previously been diagnosed by a gynecologist with infertility in a couple were informed about the possibility of taking part in a survey by a midwife, just before visiting a doctor. The invitation to the study was addressed to couples, as well as individual patients. After visiting a specialist, people who agreed to participate in the study were invited to a separate room, and they were informed by the interviewer about the purpose of the study, it’s anonymity and the right to withdraw their participation at any time without giving any reason. The completion time of the study was estimated for 20 min. Five hundred questionnaires were administered to the voluntary participants within the period June 2019 to February 2020, 472 questionnaires were returned and for the final analysis 456 fully completed questionnaires were used (91%).

### 2.2. Tools

In the paper-pencil study 3 tools were used: author’s questionnaire for the collection of socio-demographic data and medical information about respondents. Personal Information Form (PIF) and two standardized tools: Rosenberg Self-Esteem Scale (SES) and Acceptance of Illness Scale (AIS).

#### 2.2.1. Personal information form

The author’s questionnaire allowed to determine the data of socio-demographic respondents (age, sex, education status, place of residence, duration of infertility) and clinical characteristics of participants (time of treatment, type and reason of infertility).

#### 2.2.2. Self-esteem scale

To evaluate self-esteem of participants we used self-esteem scale (SES), developed by Rosenberg (43), the Polish adaptation of Łaguna et al. (53) that allows the measurement of a general level of self-esteem, which includes self-acceptance and the way one perceives oneself. It is a 10-item scale which are rated on a 4-point Likert scale (from “I definitely agree” to “I definitely disagree”). The final score is within a range from 10 to 40. A score of 10–25 points is defined as low level, 26–29 points as average level and high level 30–40 points. The Polish version of the SES has good psychometric properties, reliability measured by Cronbach’s alpha was 0.83.

#### 2.2.3. Acceptance of Illness Scale

Acceptance of Illness Scale—AIS developed by Felton, Revensson and Hinrichsen from the Center for Community Research and Action, Department of Psychology, New York University (54, 55). This scale is created to measure the disease acceptance and can be used in relation to every single illness. AIS was adapted to Polish conditions by Juczyński (56).

The AIS contains 8 statements describing the negative consequences of poor health, i.e., limitations and difficulties associated with the disease. In each statement, the respondent defines the current health situation on a five-point Liker scale (from “I definitely agree” to “I definitely disagree”). According to the key, grade 1 confirms poor adaptation to the disease, and grade 5 confirms acceptance of the disease. The sum of all points is a general measure of the degree of acceptance of the disease and ranges from 8 to 40 points. A score of <20 points is interpreted as low level, 20–30 points as a medium level and > 30 points as a high level. A high score means acceptance and adaptation to the disease and the absence of negative emotions associated with the disease. A low score means a lack of acceptance and adaptation to the disease, as well as a strong sense of social discomfort. The study used the scale in the Polish language version in Juczyński’s adaptation, Cronbach’s alpha 0.83 (56).

### 2.3. Ethical consideration

The study was conducted in accordance with the Declaration of Helsinki for medical research. Before conducting the research, the necessary approval was obtained from Bioethical Commission in Rzeszow University, Poland (resolution number: 2018/04/03).

### 2.4. Statistical analysis

Data analyses were performed using **t**he program IBM SPSS Statistics 20 (SPSS Inc., Chicago, IL, United States) was used. To verify the occurrence of differences the chi-square tests was used. Descriptive statistics were conducted to present the data: frequency (n), percentage (%), arithmetic mean (M), and standard deviation (SD). After determining the distribution (not normal), the following nonparametric tests were used to compare the variables: Mann–Whitney Test (sex, place of residence, reason of infertility), and Kruskal-Wallis Test (level of education, duration of infertility). Spearman correlations (age) were also used. A probability level (*p*) less than 0.05 was considered significant.

## 3. Results

A total of 456 patients (women and men) from infertile couples who voluntary agreed to take part in the study and who fully completed the questionnaire were included into the project. Among patients 51.5% (*n* = 235) were women and 48.5% (*n* = 221) were men. The mean age of respondents was 33.85 years (standard deviation, *SD* = 4.76, range: 20–44 years). Most respondents (*n* = 248, 54.4%) lived in the city, had a higher educational level (*n* = 295, 64.7%). The majority of men and women surveyed were 30–34 years (40.4% vs. 33.9%). The majority of women and men indicated the city as their place of residence (55.7% vs. 52.9%). Patients from infertile relationships are mostly people with higher education, both women (74.5%) and men (54.3%). Secondary education was indicated by 23.4% of women and 31.2% of men. Compared to men (13.6%), there were relatively few women with vocational education (1.5%). Most women and men indicated the time of trying to have children in the range of 3–4 years (34.0% vs. 35.7%). Detailed characteristics of respondents is shown in Table 2.

**Table 2:** Characteristic of the investigated group (*n* = 456).

Variable		Women (*n* = 235)	Men (*n* = 221)	Total (*n* = 456)
		N (%)	N (%)	N (%)
Age (years)	24–29	52 (22.1)	37 (16.7)	89 (19.5)
	30–34	95 (40.4)	75 (33.9)	170 (37.3)
	35–39	70 (29.8)	74 (33.5)	144 (31.6)
	40 and more	18 (7.7)	35 (15.8)	53 (11.6)
Location	City	131 (55.7)	117 (52.9)	248 (54.4)
	Village	104 (44.3)	104 (47.1)	208 (45.6)
Education	Primary	1 (0.4)	2 (0.9)	3 (0.7)
	Vocational	4 (1.7)	30 (13.6)	34 (7.5)
	Secondary	55 (23.4)	69 (31.2)	124 (27.2)
	University	175 (74.5)	120 (54.3)	295 (64.7)
Duration of infertility (years)	1–2	79 (33.6)	71 (32.1)	150 (32.9)
	3–4	80 (34.0)	79 (35.7)	159 (34.9)
	5–6	47 (20.0)	42 (19.0)	89 (19.5)
	7 and more	29 (12.3)	29 (13.1)	58 (12.7)
Reason of infertility	Diagnosed	122 (51.9)	110 (49.8)	232 (50.9)
	Undiagnosed	113 (48.1)	111 (50.2)	224 (49.1)

The overall self-assessment score for the whole sample is 30.50 (15 ± 30), which indicates a high level from the sample. Similarly, in the case of AIS, the result is 32.4 (8 ± 40), which also gives a high score. In the study group, men obtained a slightly higher level of self-esteem than women (31.00 vs. 30.04 points). Also, men had a higher level of acceptance of the disease (33.12 vs. 31.80) than women (Table 3).

**Table 3:** SES and AIS scores and sex of respondents.

Sex*		SES	AIS
Women	M	30.04	31.80
	SD	5.29	6.68
	Me	30.00	34.00
	Min.	15	8
	Max.	40	40
	IQR	7	10
Men	M	31.00	33.12
	SD	4.44	6.75
	Me	30.00	35.00
	Min.	20	8
	Max.	40	40
	IQR	6	10
Total	M	30.50	32.44
	SD	4.91	6.74
	Me	30.00	34.00
	Min.	15	8
	Max.	40	40
	IQR	7	10
	*p*	**0.0489**	**0.0100**

*Mann–Whitney test.

M, mean; Me, median; SD, standard deviation; Min. – minimum; Max. – maximum; IQR, interquartile range.

More than half of the respondents (55.7%) had high self-esteem (*n* = 254). Every third respondent obtained an average level of self-esteem (*n* = 152, i.e., 33.3%). Every tenth respondent (11% of people, *n* = 50) presented a low level of self-esteem.

In the study group, the majority of respondents, 71.1% of people (*n* = 324) also showed a high level of acceptance of the disease. The average (average) level of acceptance of the disease was presented by 25.0% of subjects (*n* = 114). Low acceptance of the disease was declared by 3.9% of people (*n* = 18). Every fourth respondent had an average level of acceptance of the disease.

In the further part of the study, the impact of selected socio-demographic and clinical factors on self-esteem and acceptance of the disease were analyzed. Socio-demographic variables were taken into account first: age, gender, education and place of residence.

Table 4 shows that the age of the respondents did not significantly affect the level of acceptance of the disease. However, with the age of the subjects, their self-esteem increased (rho = 0.132) (*p* = 0.0049).

**Table 4:** SES and AIS scores and age of respondents.

Age**		SES	AIS
Women	Rho	0.121	0.079
	*n*	235	235
	*p*	0.0633	0.2285
Men	Rho	0.125	0.003
	*n*	221	221
	*p*	0.0642	0.9656
Total	Rho	0.132	0.056
	*n*	456	456
	*p*	**0.0049**	0.2326

Rho, Spearman rank correlation, *n*, sample size, *p*, probability.

**Spearman’s rho coefficient tests.

The age did not significantly affect SES and AIS scores in both the female and male groups, although differences in rho values were noted, but they were not significant.

People with primary and vocational education had a reduced level of self-esteem (29.24 points) compared to people with secondary education (30.06) or higher education (30.84)—*p* = 0.0382.

Education did not significantly affect the level of disease acceptance (*p* = 0.4506). Place of residence did not significantly differentiate the level of self-esteem (*p* = 0.8913) and acceptance of the disease (*p* = 0.4974) of the surveyed patients (Table 5). There was no significant difference between SES and AIS scores in men and women according to place of residence (Table 6).

**Table 5:** SES and AIS scores and socio-demographic variables.

Place of residence*		SES	AIS
City	M	30.38	32.66
	SD	5.09	6.56
	Me	30.00	34.00
	Min.	15	11
	Max.	40	40
	n	248	248
Village	M	30.64	32.18
	SD	4.71	6.95
	Me	30.00	34.00
	Min.	18	8
	Max.	40	40
	n	208	208
Total	M	30.50	32.44
	SD	4.91	6.74
	Me	30.00	34.00
	Min.	15	8
	Max.	40	40
	n	456	456
*p*		0.8913	0.4974
Education***		SES	AIS
University	M	30.84	32.90
	SD	5.04	6.15
	Me	31.00	34.00
	Min.	15	12
	Max.	40	40
	n	295	295
Secondary	M	30.06	31.90
	SD	4.53	7.18
	Me	30.00	34.00
	Min.	16	8
	Max.	40	40
	n	124	124
Primary/Vocational	M	29.24	30.57
	SD	4.94	9.12
	Me	29.00	31.00
	Min.	19	8
	Max.	40	40
	n	37	37
Total	M	30.50	32.44
	SD	4.91	6.74
	Me	30.00	34.00
	Min.	15	8
	Max.	40	40
	n	456	456
*p*		**0.0382**	0.4506

*Mann–Whitney test. ***Kruskal-Wallis test.

M, mean; Me, median; SD, standard deviation; Min., minimum; Max., maximum; IQR, interquartile range.

**Table 6:** SES and AIS scores and socio-demographic variables by gender.

		SES		AIS	
		Woman	Man	Woman	Man
Place of residence*					
City	M	29.75	31.09	31.66	33.77
	Me	30.00	31.00	33.00	36.00
	SD	5,49	4.52	6.92	5.97
	Min.	15	21	11	12
	Max.	40	40	40	40
	IQR	7	6	9	8
Village	M	30.40	30.88	31.97	32.38
	Me	29.50	30.00	34.00	34.00
	SD	5.02	4.38	6.39	7.50
	Min.	18	20	8	8
	Max.	40	40	40	40
	IQR	7	6	10	10
*p*		0.4840	0.5021	0.9437	0.3255
Education***					
University	M	30.41	31.48	32.48	33.51
	Me	30.00	31.00	34.00	35.00
	SD	5.28	4.62	6.22	6.01
	Min.	15	21	15	12
	Max.	40	40	40	40
	IQR	7	6	10	8
Secondary	M	29.05	30.87	30.13	33.32
	Me	29.00	30.00	32.00	35.00
	SD	5.03	3.94	7.43	6.69
	Min.	16	22	8	8
	Max.	40	40	40	40
	IQR	6	6	11	10
Primary/Vocational	M	28.00	29.44	26.40	31.22
	Me	28.00	29.00	28.00	33.00
	SD	7.45	4.56	9.29	9.06
	Min.	19	20	11	8
	Max.	39	40	36	40
	IQR	13	5	14	12
*p*		0.1278	**0.0320**	**0.0397**	0.7686

*Mann–Whitney test. ***Kruskal-Wallis test.

M, mean; Me, median; SD, standard deviation; Min., minimum; Max., maximum; IQR, interquartile range. Bold *p* value = statistically significant.

Then it was checked whether the clinical situation such as the time of trying to have children, the type of infertility, the reason of infertility affected self-esteem and the level of acceptance of the disease. There were no significant differences between the duration of infertility and the level of self-esteem measured by SES (*p* = 0.7416) and the level of acceptance of disease measured by AIS (*p* = 0.4394). Respondents with secondary infertility presented a higher level of self-esteem (31.68 points) than people with primary infertility (30.26 points), *p* = 0.0162. Small differences (*p* = 0.1651) suggested that people with secondary infertility had a higher level of acceptance of the disease (33.38 points vs. 32.25 points). Knowledge of the cause of infertility did not significantly differentiate the level of self-esteem (*p* = 0.2531) or the level of acceptance of the disease (*p* = 0.9338), both in the general group and in groups women and men. Details are shown in Tables 7, 8.

**Table 7:** SES and AIS scores and clinical variables.

Duration of infertility***		SES	AIS
1–2	M	30.79	32.67
	SD	5.10	6.96
	Me	31.0	34.5
	Min.	15	11
	Max.	40	40
	n	150	150
3–4	M	30.21	32.51
	SD	5.37	5.89
	Me	30.0	34.0
	Min.	16	13
	Max.	40	40
	n	159	159
5–6	M	30.66	32.44
	SD	3.43	7.88
	Me	30.0	35.0
	Min.	23	8
	Max.	38	40
	n	89	89
7 and more	M	30.31	31.66
	SD	5.12	6.55
	Me	29.0	33.0
	Min.	20	15
	Mx.	40	40
	n	58	58
Total	M	30.50	32.44
	SD	4.91	6.74
	Me	30.0	34.0
	Min.	15	8
	Max.	40	40
	n	456	456
*p*		0.7416	0.4394
Reason of infertility*		SES	AIS
Diagnosed	M	30.72	32.65
	SD	5.04	6.27
	Me	30.0	34,0
	Min.	15	11
	Max.	40	40
	n	232	232
Undiagnosed	M	30.27	32.22
	SD	4.79	7.20
	Me	30.0	34.0
	Min.	16	8
	Max.	40	40
	n	224	224
Total	M	30.50	32.44
	SD	4.91	6.74
	Me	30.0	34.0
	Min.	15	8
	Max.	40	40
	n	456	456
*p*		0.2531	0.9338

*Mann–Whitney test. ***Kruskal-Wallis test.

M, mean; Me, median; SD, standard deviation; Min., minimum; Max., maximum; IQR, interquartile range.

**Table 8:** SES and AIS scores and clinical variables by gender.

		SES		AIS	
		Woman	Man	Woman	Man
Duration of infertility***					
1–2	M	30.59	31.00	31.97	33.44
	Me	31.00	31.00	33.00	36.00
	SD	5.60	4.50	6.83	7.08
	Min.	15	21	11	11
	Max.	40	40	40	40
	IQR	8	7	10	10
3–4	M	29.46	30.97	32.06	32.96
	Me	29.00	30.00	34.00	34.00
	SD	5.79	4.83	5.94	5.84
	Min.	16	21	15	13
	Max.	40	40	40	40
	IQR	8	7	9	9
5–6	M	30.40	30.95	32.06	32.86
	Me	30.00	30.50	34.00	35.50
	SD	3.60	3.25	7.73	8.12
	Min.	23	24	8	8
	Max.	38	38	40	40
	IQR	4	4	8	9
7 and more	M	29.52	31.10	30.17	33.14
	Me	29.00	30.00	30.00	34.00
	SD	5.29	4.91	6.46	6.40
	Min.	21	20	15	17
	Max.	40	40	40	40
	IQR	9	6	9	10
*p*		0.4934	0.9995	0.3613	0.3613
Reason of infertility*					
Diagnosed	M	30.21	31.29	32.22	33.13
	Me	30.00	31.00	34.00	34.00
	SD	5.46	4.48	6.45	6.05
	Min.	15	20	11	12
	Max.	40	40	40	40
	IQR	8	6	9	9
Undiagnosed	M	29.85	30.70	31.35	33.11
	Me	29.00	30.00	33.00	36.00
	SD	5.11	4.41	6.91	7.41
	Min.	16	21	8	8
	Max.	40	40	40	40
	IQR	6	7	9	9
*p*		0.6339	0.2238	0.3541	0.5030

*Mann–Whitney test. ***Kruskal-Wallis test.

M, mean; Me, median; SD, standard deviation; Min., minimum; Max., maximum; IQR, interquartile range.

## 4. Discussion

Infertility is a complex medical, psychological, and social problem that affects many aspects of life (57). Our study aimed to determine the factors which affect the level of self-esteem and the degree of acceptance of the disease in infertile patients.

The results of our research confirmed that the overall level of self-esteem for the studied group of patients from infertile relationships was high for the vast majority of respondents.

Previous qualitative studies have shown that infertile individuals have reduced self-esteem in comparison to the fertile group (39–41). Jamil et al. in their study observed that the self-esteem score of infertile men were significantly lower as compared to a control group of fertile men (39). Similarly, in a study Kamal et al. who concluded that infertile males had lower self-esteem than fertile males and it made them more liable to have personal as well as social problems (58). Higher self-esteem has a positive effect on the course of treatment and can mitigate the negative impact of infertility stress on depression or anxiety (15).

Zayed and El-Hadidy and Behboodi-Moghadam et al. confirm a loss of self-esteem in infertile women compared to women having children (59, 60). Cox et al. confirmed levels of self-esteem did not differ, in women whose pregnancies were the result of IVF compared to women whose pregnancies were the result of natural conception. In both groups, self-esteem increased as the pregnancy progressed. In addition, self-esteem was negatively correlated with anxiety during pregnancy, i.e., as women’s self-esteem increased, anxiety decreased (48).

The results of our study showed that self-esteem depends on sex. In the study group, women shown lower levels of self-esteem than men. Similar results were obtained by El Kissi et al. and Wischman et al., where the average self-esteem score of women was also lower than in men (10, 41). Moreover Boivin et al. observed that lower self-esteem in women was often a reaction to a diagnosis of infertility in a relationship (61). Kamal et al. identified that infertile men have lower self-esteem compared to the group of fertile men (58). Reduction in self-esteem in infertile men was also confirmed in studies by Sultan and Tahir (62), Zouari et al. (63), and Xing et al. (64).

In many countries the perception of infertility is determined by a cultural factor (22). According to current medical knowledge, infertility can be caused by a female, male, or mixed factor (2, 3). However, in many countries, it is still wrongly perceived that only the woman is responsible for infertility in a relationship (22, 33). For this reason, regardless of the cause of infertility, childless women are stigmatized, discriminated against, and excluded by the family and society (65), and men do not participate in tests confirming their fertility (22).

Our study also confirms the dependence of self-esteem on education. Those who were better educated had higher levels of self-esteem, this is consistent with Xing et al. results, although Jamil et al.’s research showed otherwise, i.e., higher self-esteem was presented by respondents with a lower level of education (39, 64).

We did not observe any significant relationship between time of infertility and self-esteem in our respondents. It was different than other studies, where the self-esteem of the respondents decreased along with the duration of infertility (39–41, 49).

According to the results of the present study, respondents also presented a high level of acceptance of the disease. Differences between men and women were also shown and the level of acceptance of the disease was higher in men. Also, people with secondary infertility had a higher level of acceptance of.

the disease, which corresponds to Dembinska’s results. In her study acceptance of infertility was also correlated with the type of infertility and lower acceptance of their own disease was observed in women with primary infertility. Dembinska confirmed that age also influenced the level of acceptance of the disease and higher acceptance by infertile women in the older age group. They also reported greater life satisfaction, a better perception of social support and a higher level of hope for the success of the treatment. The same study confirmed that higher the acceptance of the disease, lower the anxiety and depression in women (52). Our study did not confirm significant differences between patients’ age and disease acceptance.

Acceptation of infertility can also mean accepting childlessness or deciding to adopt in the long run. Patients vary in the degree of acceptance of infertility at different stages of treatment (66). Infertile women, especially in a situation where infertility lasted a long time, more easily reconciled with the facts and were willing to accept infertility treating it as their fate and destiny (36). Significant differences between men and women have also been observed in our previous studies. Only every fourth female and nearly every second male were able to accept the lack of offspring. On the other hand, more women than men declared for adoption (32). Which was consistent with Pash et al. results, where having a child was more important for women than for men (67).

As already mentioned, there are not so many studies devoted to the acceptance of one’s own infertility using AIS. The term “adjustment to infertility” appears much more often in the literature. Which can be understood as acceptance of the current situation during infertility treatment. Glover et al. explains that adapting to fertility problems is a way in which people recognize and process information about the course of their fertility problem and its treatment and possible consequences, i.e., the level of adaptation to having or not having a child in the future (66).

Better adaptation to infertility occurs in couples with greater social support and in a better financial situation (49). Study by Mahajan et al., adaptation to infertility situations was dependent on religiosity, family support, and sexual satisfaction (68). Similar dependencies are confirmed by Kroemeke and Kubicka, i.e., male gender, less social support, and shorter duration of infertility were related to better adjustment as well as with fewer symptoms of depression (69). On the other hand, Besharat et al. did not show a significant difference between men and women in terms of adjustment to infertility (70).

## 5. Limitations

Our study is based on a single observation and there was no control group, due to this reason generalization of findings is limited. Another limitation was a relatively small sample size and the fact that all the patients were recruited from only one region of Poland. Our study focused on selected factors only, so the future research could consider other variables.

## 6. Conclusion

The level of self-esteem in patients in infertile couples increases with age and the level of education. There are also significant differences between women and men, i.e., positive correlations between the level of education and self-esteem in men and the degree of acceptance of the disease in women. Clinical factors did not determine the results of SES and AIS. The results may be relevant to practitioners involved in the design and implementation of procedures addressed to couples with unexplained infertility. Interdisciplinary actions should be taken to implement therapies to strengthen self-esteem in infertile patients into infertility treatment procedures, which may contribute to reducing stress and better acceptance of one’s own infertility.

## Data availability statement

The raw data supporting the conclusions of this article will be made available by the authors, without undue reservation.

## Ethics statement

The studies involving human participants were reviewed and approved by the Bioethical Commission at Rzeszów University, Poland (resolution number: 2018/04/03). Written informed consent for participation was not required for this study in accordance with the national legislation and the institutional requirements.

## Author contributions

MN: conceptualization, writing-original draft preparation, data curation, and methodology. MN and BZ: formal analysis. MN and BO: investigation. MN, BZ, and DD-K: writing—review and editing. MN, BO, and DD-K: supervision. All authors have read and agreed to the published version of the manuscript.

## Conflict of interest

The authors declare that the research was conducted in the absence of any commercial or financial relationships that could be construed as a potential conflict of interest.

## Publisher’s note

All claims expressed in this article are solely those of the authors and do not necessarily represent those of their affiliated organizations, or those of the publisher, the editors and the reviewers. Any product that may be evaluated in this article, or claim that may be made by its manufacturer, is not guaranteed or endorsed by the publisher.
